# Herbivory-induced systemic signals are likely to be evolutionarily conserved in euphyllophytes

**DOI:** 10.1093/jxb/erab349

**Published:** 2021-07-22

**Authors:** Yunting Lei, Yuxing Xu, Jingxiong Zhang, Juan Song, Jianqiang Wu

**Affiliations:** 1 Department of Economic Plants and Biotechnology, Yunnan Key Laboratory for Wild Plant Resources, Kunming Institute of Botany, Chinese Academy of Sciences, Kunming, China; 2 CAS Center for Excellence in Biotic Interactions, University of Chinese Academy of Sciences, Beijing, China; 3 The James Hutton Institute, UK

**Keywords:** Dodder, evolution, herbivory, jasmonic acid, systemic signaling, vascular plants

## Abstract

Herbivory-induced systemic signaling has been demonstrated in monocots and dicots, and is essential for plant defense against insects. However, the nature and evolution of herbivory-induced systemic signals remain unclear. Grafting is widely used for studying systemic signaling; however, grafting between dicot plants from different families is difficult, and grafting is impossible for monocots. In this study, we took advantage of dodder’s extraordinary capability of parasitizing various plant species. Field dodder (*Cuscuta campestris*) was employed to connect pairs of species that are phylogenetically very distant, ranging from fern to monocot and dicot plants, and so determine whether interplant signaling occurs after simulated herbivory. It was found that simulated herbivory-induced systemic signals can be transferred by dodder between a monocot and a dicot plant and even between a fern and a dicot plant, and the plants that received the systemic signals all exhibited elevated defenses. Thus, we inferred that the herbivory-induced systemic signals are likely to be evolutionarily well conserved among vascular plants. Importantly, we also demonstrate that the jasmonate pathway is probably an ancient regulator of the biosynthesis and/or transport of systemic signals in vascular plants. These findings provide new insight into the nature and evolution of systemic signaling.

## Teaser

Ferns, monocots, and dicots use the same signals to enhance defense responses in non-damaged leaves when a leaf is attacked by insects.

## Introduction

Different plant parts often suffer from localized stresses. For example, a region of a leaf or root may be challenged by insect feeding or pathogen infection, and a portion of a root could experience phosphate deficiency, because of uneven distribution of soil phosphate. As an integrated system, plants are able to perceive localized stresses and respond with whole plant-wide physiological responses. These responses in the distal systemic tissues or organs are considered to be mediated by certain systemic signals produced in the local stress-inflicted tissue or organ (Fichman and Mittler, 2020). The systemic signals enable communications between different parts of plants, and this process is important for coordinating responses to stresses on a whole-plant level. There are at least three types of systemic acclimation or responses: systemic acquired resistance to pathogens; systemic wound response (SWR) to damage and herbivory; and systemic acquired acclimation to abiotic stresses (Hilleary and Gilroy, 2018).

SWR is considered to be an important part of a plant’s sophisticated defense responses to insect feeding (Schuman and Baldwin, 2016; Erb and Reymond, 2019). For example, after simulated insect herbivory on wild tobacco (*Nicotiana attenuata*), elevated defenses were not only detected at the site of damage, but also in the other parts of the damaged (local) leaf and in the undamaged (systemic) leaves (Wu *et al.*, 2007; Hettenhausen *et al.*, 2014). SWR was first discovered in tomato (*Solanum lycopersicum*) almost 50 years ago (Green and Ryan, 1972). However, the nature of the mobile systemic signals that activate SWRs remains unclear. Increasing lines of evidence have revealed that in dicotyledonous species serval signals, including jasmonic acid (JA) (Li *et al.*, 2002; Koo *et al.*, 2009), reactive oxygen species and Ca^2+^ (Miller *et al.*, 2009; Kiep *et al.*, 2015; Toyota *et al.*, 2018), and electric signals (Hedrich *et al.*, 2016; Zimmermann *et al.*, 2016), play important roles in wounding/herbivory-induced systemic signaling. Moreover, SWRs have also been detected in monocots. For example, simulated lepidopteran insect *Mythimna separata* feeding and wounding on maize (*Zea mays*) leaves induced JA and JA-isoleucine conjugate (JA-Ile) in the systemic leaves, and simulated herbivory, but not mechanical wounding, specifically activated accumulation of the defensive metabolites benzoxazinoids (Bxs) systemically (Malook *et al.*, 2019). The western corn rootworm (*Diabrotica virgifera virgifera*), a major pest of maize, exhibited increased avoidance of maize roots if the leaves had been infested by the caterpillar *Spodoptera littoralis*, indicating leaf to root systemic signaling in maize (Erb *et al.*, 2015).

It is estimated that 450–430 million years ago, the common ancestor of vascular plants evolved from bryophytes (mosses, hornworts, and liverworts), and gradually formed lycophytes and euphyllophytes, including ferns, gymnosperms, and angiosperms (monocots and dicots) (Kenrick, 2000; Tomescu *et al.*, 2014; Morris *et al.*, 2018). The vascular system plays an essential role in transporting water and nutrients between different plant parts. Importantly, providing interconnection between distant organs, the vasculature has also long been recognized as an information superhighway (Lucas *et al.*, 2013), in which numerous biomolecules ranging from proteins and RNAs to hormones and metabolites are carried by the flow of the transpiration and translocation streams. Even though little is known about the nature of systemic signals, the plant vascular systems are thought to be the most important conduits for translocation of systemic signals (Heil and Ton, 2008; Shah, 2009). To date, however, whether wounding- and/or herbivory-induced systemic signals that activate SWRs are conserved in all vascular plants remains unclear.

Grafting has long been used to study systemic signaling in plants (Gaut *et al.*, 2019). However, scion and rootstock mostly need to be plants from the same family, as interfamily grafting is very difficult or even impossible (Goldschmidt, 2014). Grafting in monocots is not possible due to lack of cambium tissue. Dodders (*Cuscuta*, Convolvulaceae) are widely distributed parasites. These parasites are leafless and rootless, and their stems twine around host stems. Along dodder stems many haustoria are formed; the haustoria penetrate the host stem, and dodder haustorial phloem and xylem are respectively fused with the phloem and xylem of the host stem, enabling dodder not only to extract water and nutrients, but also to obtain host secondary metabolites, proteins, and RNAs (Clarke *et al.*, 2019). The parasitization process could be considered to be somewhat similar to artificial grafting. Many dodder species have very wide ranges of hosts. An individual dodder plant can simultaneously parasitize two or multiple adjacent hosts from the same or different families, forming a dodder-connected plant cluster. The dodder’s unique capability of parasitizing two or more hosts provides a very convenient system for studying systemic signaling even between phylogenetically distant species. Recently, systems composed of different dodder-connected dicot hosts have been used to study wounding-, insect feeding-, salt-, and nutrient stress-induced systemic signaling, and it was found that these biotic and abiotic stress-induced systemic signals can be transferred across different species and activate strong physiological changes in the recipient plants (Hettenhausen *et al.*, 2017; Qin *et al.*, 2019; Li *et al.*, 2020; Zhang *et al.*, 2021). For example, in a system in which an Arabidopsis (*Arabidopsis thaliana*) plant and a tobacco (*Nicotiana tabacum*) plant were bridge-connected by a dodder *Cuscuta australis*, wounding the Arabidopsis strongly elevated the activity of trypsin proteinase inhibitors (defensive metabolites) in tobacco; importantly, when the wild-type Arabidopsis was replaced by the mutant *dde2-2*, which is impaired in JA biosynthesis, wounding *dde2-2* could no longer activate tobacco defenses; thus, wounding-/herbivory-induced systemic signals are commonly shared in dicots, and JA plays an important role in regulating the production or transport of the systemic signals (Hettenhausen *et al.*, 2017).

In this study, taking advantage of dodders’ extraordinary capability of connecting different plants, we used the field dodder *Cuscuta campestris* to attach to different vascular plants, ranging from ferns to monocot and dicot plants. We show that herbivory-induced systemic signals are well conserved among monocots, dicots, and even ferns, and the plants which received the systemic signals all exhibited elevated defense responses and resistance to insects. Furthermore, using Arabidopsis and maize mutants impaired in JA biosynthesis, we show that JA seems to play a conserved role in regulating systemic signals in vascular plants.

## Materials and methods

### Plant materials and growth conditions

Maize (*Zea mays* cv. W22) and the maize *lox8* mutant (W22 background) (Acosta *et al.*, 2009), spring onion (*Allium ascalonicum*), Arabidopsis (*Arabidopsis thaliana* Col-0) and the *dde2-2* mutant (Col-0 background) (von Malek *et al.*, 2002), sword fern (*Nephrolepis cordifolia*), and tobacco (*Nicotiana tabacum* cv. Samsun) were grown in a glasshouse maintained at ~25 °C (day)/18 °C (night) and 14 h light/10 h dark. Dodder (*Cuscuta campestris*) seedlings were infested on soybean (*Glycine max*) plants to form stocks. Freshly excised dodder stem segments from vigorously growing dodder stocks, 5–10 cm in length, were used to infest Arabidopsis, spring onion, or tobacco host plants. Approximately 2 weeks after initial attachment, new stems emerged and elongated, and the elongated stems were manually guided to infest the neighboring plants to establish the plant clusters Arabidopsis–maize, spring onion–maize, or tobacco–sword fern (dodder bridge connections). To rule out the effect of airborne signals, in the plant clusters Arabidopsis–maize, spring onion–maize, or tobacco–sword fern, the dodders were snipped before further W+OS treatment on the host plants (see below).

### Plant treatment

*Spodoptera litura* eggs were supplied by Keyun Biocontrol (http://www.keyunnpv.cn/). For collecting *S. litura* oral secretions (OS), larvae (third to fifth instar) were reared on tobacco, and OS were collected on ice with a pipette and were immediately divided into small aliquots before being stored at –80 °C. For each plant cluster, to simulate *S. litura* herbivory, the leaves of one host plant were wounded with a pattern wheel, and 40 μl of *S. litura* OS were gently rubbed into the fresh puncture wounds (W+OS treatment); if wounding treatment was needed, plants were similarly wounded with a pattern wheel, and 40 μl of water was gently rubbed into the fresh puncture wounds (W+W treatment). At 3 h and/or 72 h after the treatment, the other intact host plant’s leaves were respectively harvested for the analysis of herbivory-elicited changes in the transcriptome and/or secondary metabolites.

### Spodoptera litura growth assay

In Arabidopsis–maize, spring onion–maize, or sword fern–tobacco plant clusters, the signal sender plants’ leaves (Arabidopsis, spring onion, or sword fern) were treated with simulated herbivory (treatment group) or kept untreated (control group), and, after 72 h, freshly hatched *S. litura* neonates were infested on the signal receiver plants (six larvae per maize or tobacco plant, 10 replicates for each type of plant cluster in either group), and their masses were recorded over time.

### Transcriptome analysis

Three biological replicates were used for library constructions and sequencing. Each sample was initially treated with Fruit-Mate for RNA purification (Takara) and thereafter the total RNA was isolated with TRIzol Reagent (Thermo Fisher Scientific). An Illumina TruSeq RNA Sample Prep Kit (Illumina) was used to build cDNA libraries. The generated cDNA libraries were sent to a HiSeq2500-PE125 platform (Illumina) to obtain sequence reads. Based on the genome sequences of maize (B73, RefGen_v4), the Tophat and Cufflinks packages were used to assemble the transcripts and to identify differentially expressed genes (DEGs) using DEGseq2 (Trapnell *et al.*, 2012; Love *et al.*, 2014), and only genes whose levels were at least 2-fold changed with statistical significance (false discovery rate <0.05) were considered to be DEGs. Gene Ontology (GO) analysis and graphing were performed on Omicshare (https://www.omicshare.com/tools/), ImageGP (http://www.ehbio.com/), and agriGO (http://systemsbiology.cau.edu.cn/agriGOv2).

### Quantification of benzoxazinoids, glucosinolates, and trypsin proteinase inhibitor activity

At least five biological replicates were analyzed. Contents of benzoxazinoids in maize (Glauser *et al.*, 2011) and glucosinolates in Arabidopsis (Burow *et al.*, 2006), and the activity of trypsin proteinase inhibitor (TPI) in tobacco (Jongsma *et al.*, 1994) were analyzed following the previously described methods.

## Results

### Simulated herbivory induces systemic signaling between dodder-bridged dicots and monocots

Most monocotyledonous species are not hosts for dodders, except that dodders are able to parasitize the monocotyledonous *Allium* plants and even complete its life cycle (Lanini and Kogan, 2005). Furthermore, we found that if a dodder initially grows on a compatible dicot host (e.g. Arabidopsis) or an *Allium* host, the elongated dodder stems can form a stable connection with the neighboring maize plant, a monocotyledonous species (Supplementary Fig. S1). Thus, dodder can serve as a natural agent that connects dicots with monocots, and monocots with monocots, enabling study of the systemic signal transfer between evolutionarily very distant plants.

**Fig. 1.**
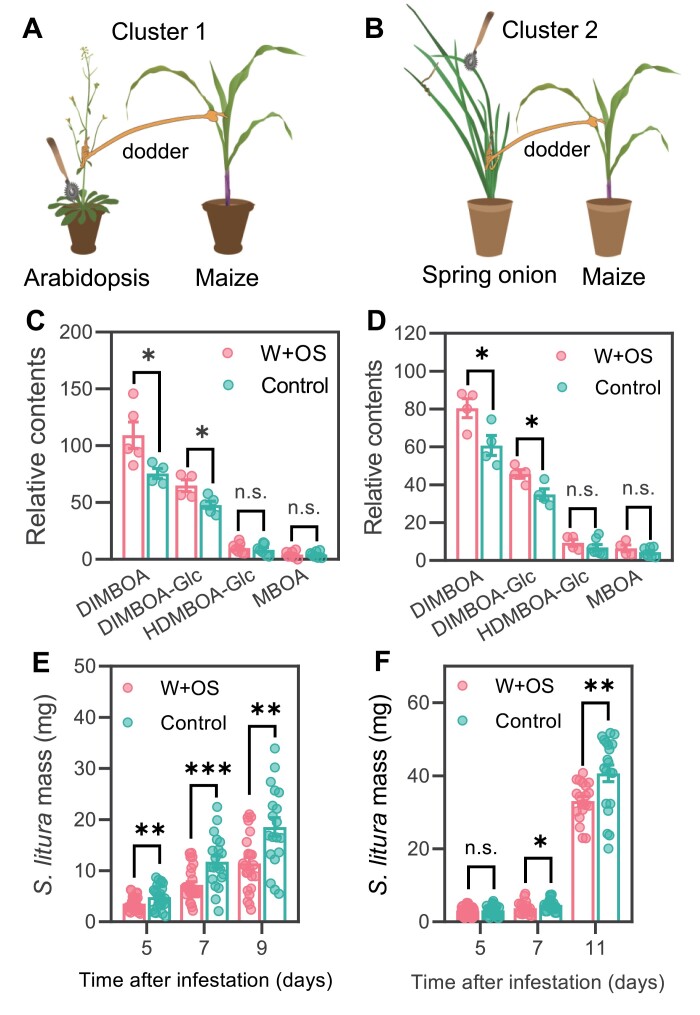
Dodder *C. campestris* conveys simulated herbivory-induced systemic signals from Arabidopsis to maize and from spring onion to maize. The schematics of Cluster 1 (Arabidopsis–maize) (A) and Cluster 2 (spring onion–maize) (B). The signal senders Arabidopsis and spring onion were treated with W+OS or were untreated (control) and, after 72 h, the respective signal receivers (maize) were harvested for determination of relative Bx contents in maize of Cluster 1 (C) and Cluster 2 (D) (*n*=6). The signal sender plants were treated with W+OS or untreated (control); after 72 h, *S. litura* larvae were infested on the signal receiver plants. The masses of insects grown on maize of Cluster 1 (E) and Cluster 2 (F) were recorded at the indicated times (*n*=19–60). Data are means ±SE, and the asterisks indicate significant differences between the control and W+OS-treated group (*P<0.05, **P<0.01, and ***P<0.001, *t*-test; n.s.=non-significant).

Taking advantage of this unique system, plant clusters of two combinations were established: Cluster 1 Arabidopsis–maize (dodder bridge connections) (Fig. 1A) and Cluster 2 spring onion (*A. ascalonicum*)–maize (Fig. 1B). In these plant clusters, Arabidopsis and spring onion were assigned to be the systemic signal senders: their leaves were either untreated (control group) or were wounded with a pattern wheel and *S. litura* OS were applied to wounds (W+OS treatment group). To examine whether the respective signal receiver plants responded to the systemic signals, the leaves of maize from Cluster 1 and 2 were harvested 72 h after the treatment on the signal senders for quantification of defensive metabolites against insects. W+OS treatment on the signal sender Arabidopsis induced 45% and 36% increased levels of the Bxs 2,4-dihydroxy-7-methoxy-2H-1,4-benzoxazin-3(4H)-one (DIMBOA) and 2,4-dihydroxy-7-methoxy-2H-1,4-benzoxazin-3(4H)-one-glucoside (DIMBOA-Glc) in the signal receiver maize of Cluster 1 (Fig. 1C); similarly, the contents of DIMBOA and DIMBOA-Glc, respectively, increased 32% and 31% in the maize plants of Cluster 2 (Fig. 1D). Next, we determined if the signal receivers have increased resistance to insects. Three days after W+OS treatment on the respective signal senders, the signal receiver plants were infested with *S. litura* larvae and their masses were recorded over time. Indeed, after the Arabidopsis was treated with W+OS, *S. litura* larvae on the maize plants of Cluster 1 were 27, 39, and 38% smaller at 5, 7, and 9 d after infestation, compared with those infested on maize plants of the control group (Fig. 1E). Similarly, in Cluster 2, W+OS treatment on the spring onions reduced the average mass of *S. litura* grown on maize 25% and 23%, respectively, on days 7 and 11 (Fig. 1F).

**Fig. 2.**
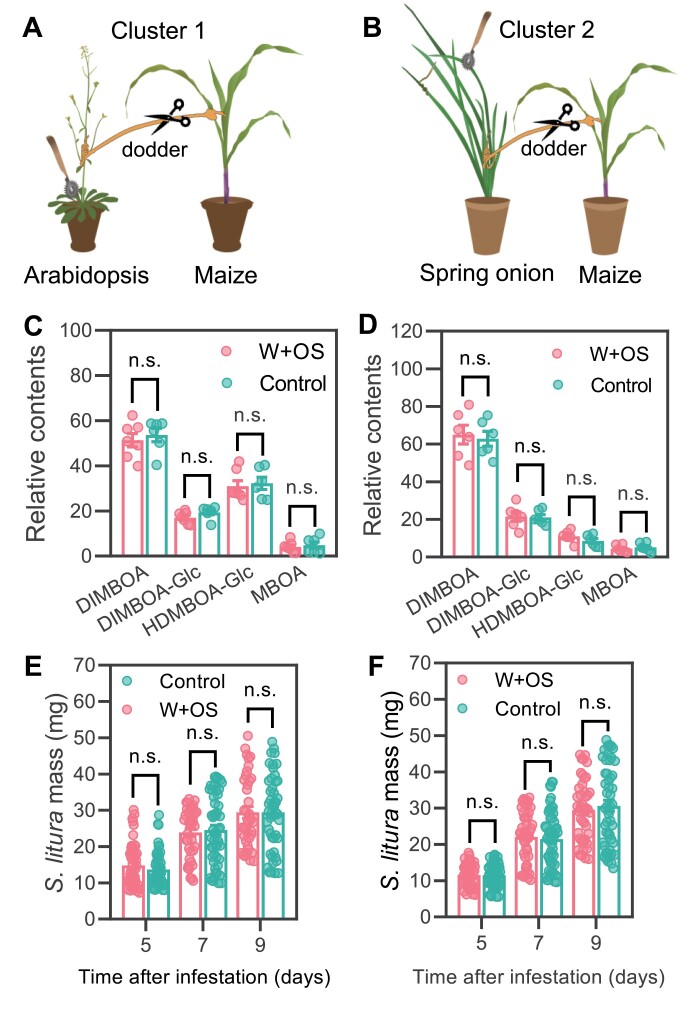
Simulated herbivory-induced airborne signals from one plant do not induce defenses in the other neighboring plant. (A and B) The schematics of plant settings. Cluster 1 (A) and Cluster 2 (B) were arranged similarly to those indicated in Fig. 1A and B; next, the connecting dodders were snipped and the signal senders Arabidopsis and spring onion plants were treated with W+OS or untreated (control); after 72 h, the respective signal receiver maize plants in Cluster 1 (C) and Cluster 2 (D) were harvested for determination of relative Bx contents (*n*=7). (E and F) Resistance of signal receiver plants to *S. litura*. The Arabidopsis and spring onion plants were treated with W+OS or untreated (control); after 72 h, *S. litura* larvae were infested on the maize plants and the masses of these insects were recorded at the indicated times in Cluster 1 (E) and 2 (F) (*n*=39–57). Data are means ±SE, and the asterisks indicate significant differences between control and the W+OS-treated group (*t*-test; n.s.=non-significant).

Some plant volatile compounds can function as airborne long-distance signals activating defense-related responses in the neighboring plants (Heil and Ton, 2008). To rule out the involvement of airborne signals from signal senders in activating defenses and resistance in the signal receivers, Cluster 1 and Cluster 2 were arranged similarly to Cluster 1 and 2 in Fig. 1, respectively; next, the connecting dodders were snipped and the signal senders (Arabidopsis and spring onion) were immediately treated with W+OS or untreated, and the signal receiver maize plants were harvested after 72 h (Fig. 2A, B), which were used to analyze defensive metabolites and resistance to *S. litura*. As expected, the data of defensive metabolites (Fig. 2C, D) and the growth of *S. litura* (Fig. 2E, F) indicated that the airborne signals, if there were any, did not have detectable effects on the neighboring signal receivers.

The interplant systemic signaling was also confirmed by reversing the signal senders and receivers in Cluster 1 and 2. In Cluster 1 (Supplementary Fig. S2A), 3 d after W+OS treatment on the maize leaves, the defensive glucosinolates indole-3-ylmethyl-glucosinolate (I3M), 4-methoxy-indole-3-ylmethyl-glucosinolate (4MO-I3M), and 1-methoxy-indole-3-ylmethyl-glucosinolate (1MO-I3M) in the Arabidopsis plants were respectively 34, 62, and 29% greater than in the Arabidopsis plants of the control group (Supplementary Fig. S2B). In Cluster 2 (Supplementary Fig. S2C), 3, 6, and 12 d after W+OS treatment on the maize leaves, the masses of *S. litura* larvae were also respectively 39, 27, and 23% decreased on the spring onion plants, compared with those infested on the spring onion plants of the control group (Supplementary Fig. S2D). Thus, W+OS treatment on monocotyledonous and dicotyledonous signal senders can activate defense-related responses in signal receiver plants, regardless of whether the receiver is a monocot or dicot.

**Fig. 3.**
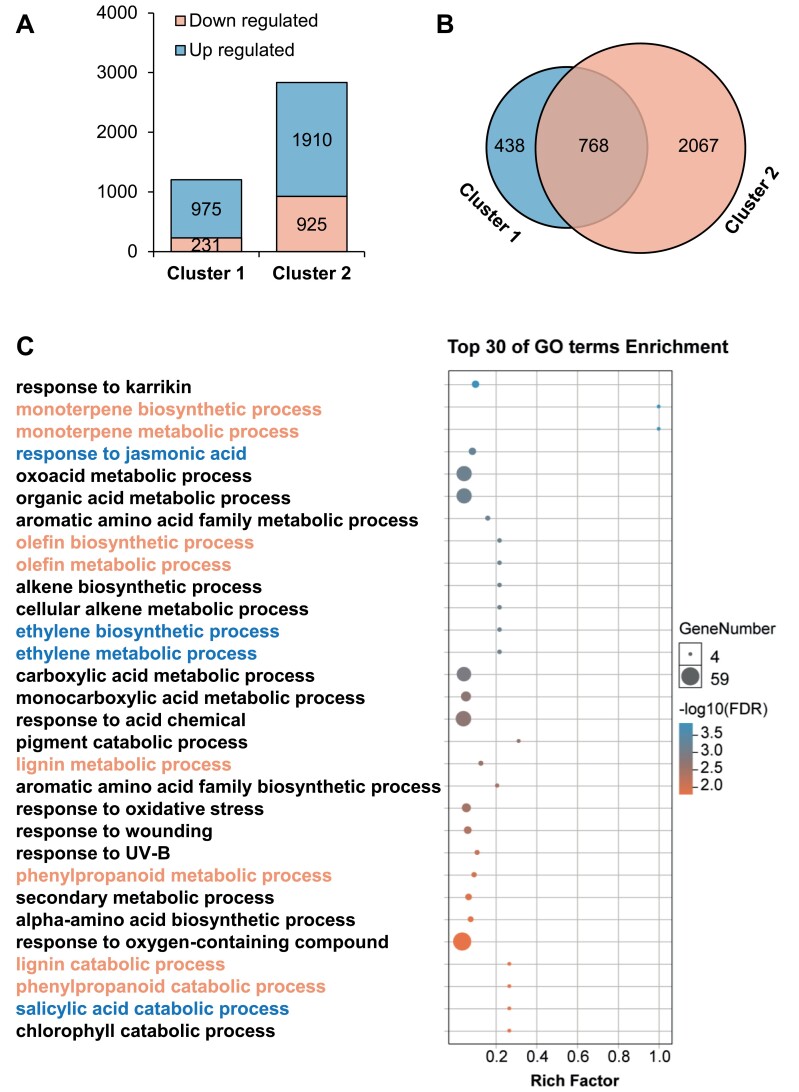
Simulated herbivory on Arabidopsis in Cluster 1 and spring onion in Cluster 2 elicits somewhat similar transcriptomic changes in maize plants. In Cluster 1 (Arabidopsis–maize) and Cluster 2 (spring onion–maize), the signal sender plants (Arabidopsis and spring onion) were treated with W+OS or not treated (control); after 3 h, the signal receiver plants were harvested for RNA-seq. (A) The numbers of DEGs in the maize plants of Cluster 1 and 2. (B) Venn diagram indicating the specific and common DEGs in the maize of Cluster 1 and 2. (C) The top 30 GO terms enriched from the commonly regulated 768 DEGs in the maize of Cluster 1 and 2. Dark blue, GO terms related to phytohormone pathways; pale orange, GO terms related to secondary metabolic processes.

To gain further insight into dodder-mediated interplant systemic signaling, Arabidopsis and spring onion plants in Clusters 1 and 2 were treated with W+OS or left untreated (control), and, after 3 h, the maize leaves from both clusters, which received the systemic signals (in the W+OS group) or not (in the control group) were harvested for RNA-sequencing (RNA-seq) analysis. Using the RNA-seq data of maize leaves from the respective control groups as the baselines, the DEGs in the maize leaves of Cluster 1 and 2 were obtained. In Cluster 1, W+OS treatment on Arabidopsis resulted in 1206 DEGs (975 up- and 231 down-regulated) in the maize plants; in Cluster 2, 2835 DEGs (1910 up- and 925 down-regulated) were identified in the maize plants after W+OS treatment on the spring onion plants (Fig. 3A; Supplementary Tables S1, S2). These RNA-seq data further corroborate that dodder bridge connections allow transfer of herbivory-induced signals between dicots and monocots and between different monocots. Importantly, 768 DEGs were co-regulated in the maize plants of these two plant clusters (Fig. 3B; Supplementary Table S3), and GO analysis on these co-regulated genes indicated enrichment in stress-related phytohormone pathways (Fig. 3C; Supplementary Table S4), such as ‘response to jasmonic acid’, ‘ethylene metabolic’, and ‘salicylic acid catabolic’, and secondary metabolic processes including ‘phenylpropanoid metabolic’, ‘lignin metabolic’, ‘olefin metabolic’, and ‘monoterpene metabolic’, which are often involved in plant response to wounding or insect feeding (Dicke and Baldwin, 2010; Erb *et al.*, 2012; Liu *et al.*, 2018). Specifically, as depicted by a heatmap analysis, several genes involved in the JA pathway were among the up-regulated genes in both of the maize plants of Cluster 1 and 2, including *4-coumarate-CoA ligase-like* 5 (*4CLL5/OPCL1*; Zm00001d027519), *allene oxide synthase* (*AOS*; Zm00001d034186 and Zm00001d013185), *lipoxygenase* (*LOX*; Zm00001d027893 and Zm00001d033300), *allene oxide cyclase* (*AOC*; Zm00001d029594), *12-oxo-phytodienoic acid reductase* (*OPR*; Zm00001d003584), *jasmonic acid-amido synthetase* (*JAR1*; Zm00001d011377), and *jasmonate ZIM domain-containing protein* 3 (*JAZ3/TIFY 6B*; Zm00001d005726) (Supplementary Fig. S3A; Supplementary Table S5). Similarly, several genes involved in salicylic acid, ethylene, and abscisic acid biosynthesis and/or signal pathways were also up-regulated in both of the maize plants of Cluster 1 and 2 (Supplementary Fig. S3A; Supplementary Table S5), such as *1-aminocyclopropane-1-carboxylate oxidase* (*ACO*; Zm00001d024843, Zm00001d024851, and Zm00001d024853), *1-aminocyclopropane-1-carboxylate synthase* (*ACS*; Zm00001d026060), and *phenylalanine ammonia lyase* (*PAL*; Zm00001d017275, Zm00001d017276, Zm00001d017279, and Zm00001d051163). Furthermore, consistent with the changes of Bx contents in maize, the expression of the biosynthetic genes *BX1–BX14*, which encode the enzymes catalyzing the biosynthesis of Bxs in maize (Zhou *et al.*, 2018), were induced in maize plants of Cluster 1 and 2 (Supplementary Fig. S3B; Supplementary Table S6). These transcriptome data suggest that the systemic signals from Arabidopsis and spring onion at least partly activated somewhat similar transcriptome changes in the signal receiving maize plants.

### Simulated herbivory on sword fern activates responses in tobacco plants

Ferns are ancient vascular plants, and they are non-hosts for dodder parasites. We found that if a dodder initially grows on a compatible dicot host (e.g. tobacco), the elongated dodder stems can stably connect with the neighboring sword fern plant (*N. cordifolia*) (Supplementary Fig. S4). Such a sword fern–tobacco system can be used to study systemic signaling between dicots and ferns.

Ferns respond to insect feeding (Imbiscuso *et al.*, 2009; Radhika *et al.*, 2012). To study whether sword fern *N. cordifolia* specifically responds to simulated herbivory, we first treated *N. cordifolia* with wounding or simulated herbivory and analyzed the changes of JA and JA-Ile levels. In sword fern, wounding (W+W) and W+OS treatment both induced the contents of JA and JA-Ile (Supplementary Fig. S5); in particular, W+OS-induced JA-Ile is >7-fold that of W+W treatment (Supplementary Fig. S5B). Hence, sword fern is able to perceive certain components in the *S. litura* OS and activate the JA pathway.

**Fig. 4.**
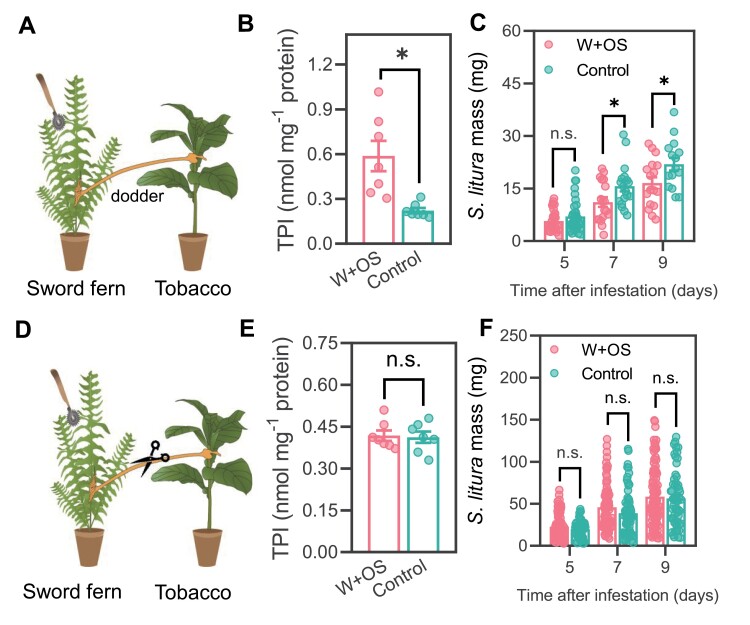
Simulated herbivory-induced systemic signals from sword ferns were conveyed by dodder to the tobacco plants, but not by airborne signals. (A–C) Responses of tobacco to dodder-transmitted signals from sword fern. (A) Schematics of plant cluster sword fern–tobacco. (B) Defensive metabolites in the signal receiver plants. The signal sender sword ferns were treated with W+OS or untreated (control); after 72 h, the signal receiver tobacco plants were harvested for determination of TPI activity (*n*=7). (C) Resistance of signal receiver plants to *S. litura*. The signal sender plants were treated with W+OS or untreated (control); after 72 h, *S. litura* larvae were infested on the signal receiver plants and the masses of these insects were recorded at the indicated times (*n*=15–37). (D–F) Responses of tobacco to airborne signals from sword fern. (D) Schematics of plant cluster and treatment. Sword fern and tobacco plants were set up similarly to those indicated in (A), and the connecting dodders were snipped before W+OS treatment on sword fern. (E) Defensive metabolites in the signal receiver tobacco plants. The signal sender sword ferns were treated with W+OS or untreated (control); after 72 h, the respective signal receiver tobacco plants were harvested for determination of TPI activity (*n*=7). (F) Resistance of signal receiver tobacco plants to *S. litura*. The sword ferns were treated with W+OS or untreated (control); after 72 h, *S. litura* larvae were infested on the tobacco plants and the masses of these insects were recorded at the indicated times (*n*=51–59). Data are means ±SE, and the asterisks indicate significant differences between the control and W+OS-treated group (*P<0.05, *t*-test; n.s.=non-significant).

Next, we set up plant clusters, each of which was composed of a dodder-connected sword fern and tobacco plant (Fig. 4A). The sword ferns were designated as the systemic signal senders, which were untreated (control group) or treated with simulated herbivory (W+OS treatment group). After 72 h, the TPI activity in the respective signal receiver tobacco was quantified. It was found that the tobacco of the W+OS treatment group had 1.6-fold greater TPI activity than did the tobacco of the control group (Fig. 4B). Consistently, treating the sword ferns with W+OS also increased the resistance of tobacco, as 7 d and 9 d after *S. litura* infestation on tobacco the average mass of *S. litura* was 36% and 24% smaller, compared with that of *S. litura* larvae infested on the tobacco of the control group (Fig. 4C). Moreover, to rule out the possibility that airborne signals from sword fern activated the defense and resistance in the tobacco plants, the connecting dodders between sword fern and tobacco were snipped (Fig. 4D), and we found that treating the sword ferns with W+OS or not did not affect the TPI activity in tobacco (Fig. 4E) or the growth of *S. litura* (Fig. 4F) on the tobacco plants, confirming that the systemic signals from sword fern were conveyed by the dodder to the tobacco, not by airborne signals, if indeed there were any. There are no defensive metabolites known in the sword fern *N. cordifolia*, and no *N. cordifolia*-feeding insects were available. Hence, the systemic response of sword fern to treatment on tobacco was not examined. Given that W+OS treatment on sword fern activated defense-related responses in tobacco (Fig. 4B, C), perception of systemic signals produced in monocots or dicots by ferns is very likely.

Thus, herbivory-induced systemic signals produced by sword fern, an evolutionarily ancient vascular plant, can be perceived by dicotyledonous tobacco, suggesting that the systemic signals are likely to be very well conserved in vascular plants.

### JA pathway plays a conserved role in regulating herbivory-induced systemic signals

**Fig. 5.**
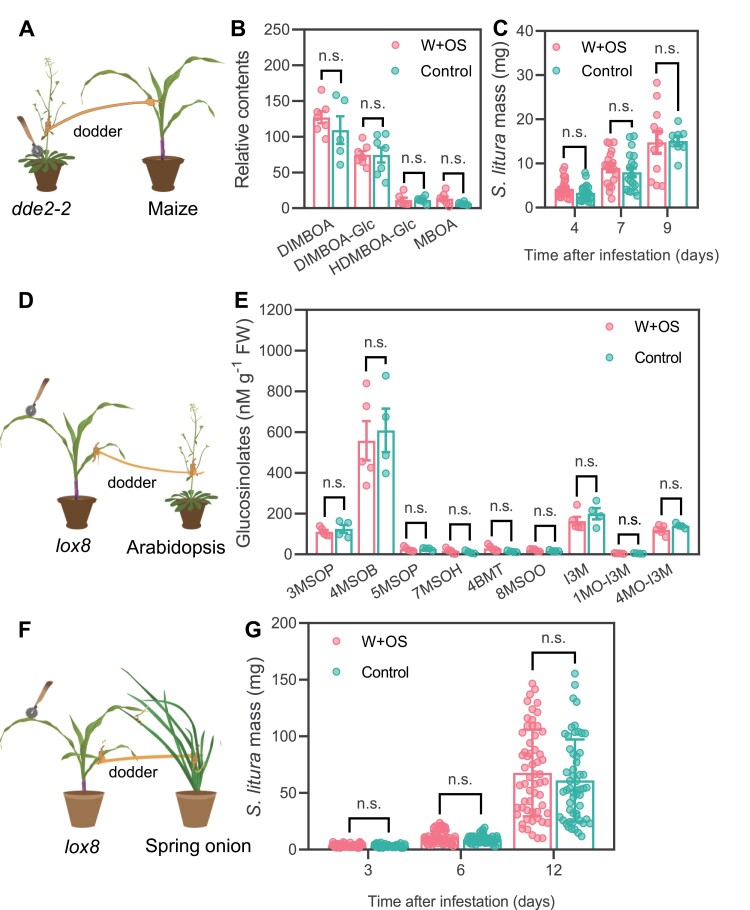
The JA pathway is required for herbivory-induced interplant signaling between phylogenetically very distant plants. In the *dde2-2*–maize plant cluster (A), the *dde2-2* mutants were treated with W+OS or untreated (control); after 72 h, the maize leaves (*n*=6) were harvested for quantification of benzoxazinoids (B) or were infested with *S. litura* and insect masses were recorded on days 4, 7, and 9 (C; *n*=8–26). Contents of glucosinolates in Arabidopsis after treating the maize *lox8* mutant with W+OS or without treatment (control). The experimental set-up is illustrated in (D); 72 h after treatment, the Arabidopsis leaves (*n*=6) were harvested for analysis of glucosinolates (E). The experimental set-up is shown in (F); 72 h after treatment, the spring onions were infested with *S. litura* larvae and their masses were recorded (G; *n*=44–59). The values are means ±SE (*t*-test; n.s.=non-significant).

In dicotyledonous plants, JA has been recognized to be important for wounding/herbivory-induced systemic signaling, even though JA itself is unlikely to be the mobile signal (Li *et al.*, 2002; Koo *et al.*, 2009; Hettenhausen *et al.*, 2017). However, whether the JA pathway is essential for systemic signaling in other vascular plants, including monocots and ferns, was unknown. To this end, we used an Arabidopsis mutant, *dde2-2* (von Malek *et al.*, 2002), and a maize mutant, *lox8*/*ts1* (Acosta *et al.*, 2009), whose JA biosynthetic genes *AOS* and *LOX8* were respectively knocked out, to create dodder-bridged plant clusters, in which these JA mutants were designated as the signal senders. In the *dde2-2*–maize clusters (Fig. 5A), *dde2-2* mutants were treated with W+OS or kept untreated (control), and the maize leaves were harvested 72 h after treatment. Quantification of Bxs showed that W+OS treatment on *dde2-2* could no longer activate the accumulation of Bxs in maize (Fig. 5B) and, consistently, *S. litura* growth was not affected by the treatment of W+OS on the *dde2-2* mutant (Fig. 5C). In the *lox8*–Arabidopsis clusters (Fig. 5D), the contents of glucosinolates in the Arabidopsis plants exhibited no obvious changes either, regardless of whether the *lox8* maize mutant was treated with W+OS or untreated (Fig. 5E). Similarly, unlike the wild-type maize, W+OS treatment on the *lox8* maize mutants in the *lox8*–spring onion clusters (Fig. 5F) could not enhance the resistance of spring onion to *S. litura* (Fig. 5G). Thus, these data suggest that the JA pathway is required for the production and/or movement of herbivory-induced systemic signals in both monocots and dicots.

## Discussion

Previously, using plant clusters composed of dodder-connected dicotyledonous hosts from different families, it has been shown that the herbivory-induced systemic signals are conserved among dicots (Hettenhausen *et al.*, 2017). In this study, we show that if a dodder initially grows on a compatible dicotyledonous or monocotyledonous (i.e. *Allium* species) plant, the elongated dodder stems can connect with adjacent plants, including monocot, dicot, and even fern species (Supplementary Figs S1, S4). The dodder’s unique capability of connecting phylogenetically very distant plants enabled us to examine whether herbivory-induced systemic signals are evolutionarily conserved in different vascular plants. Our data indicate that the systemic signals can be produced in monocots, dicots, and even sword fern, and, after being transferred by dodder (dicot), the systemic signals can be perceived by monocots and dicots (and probably ferns), resulting in induced responses and enhanced resistance in the signal recipient plants (Figs 1–4; Supplementary Figs S2, S3; Supplementary Tables S1–S6). Lycophytes represent the extant most ancient vascular plants, and gymnosperms evolved prior to angiosperms (Bowman *et al.*, 2017). Species of lycophytes or gymnosperms were not included in this study, since we did not find suitable lycophytes or gymnosperms for dodder connection. Nevertheless, it is still plausible to speculate that herbivory-induced systemic signaling is well conserved in vascular plants: the co-evolution between plants and insects started >350 million years ago (Gatehouse, 2002), a time when vascular plants started to appear (Morris *et al.*, 2018), and it is possible that the selection pressure from insect herbivory drove the evolution of systemic signaling in the common ancestor of vascular plants. Whether the systemic signals are conserved in lycophytes and gymnosperms remains to be studied, and it is unclear whether dodder is able to modify or transform the systemic signals to obtain certain ecological benefit; even though our data did not show any evidence supporting this scenario, this possibility cannot be completely ruled out.

A grafting experiment using wild-type tomato and tomato mutants impaired in JA biosynthesis and perception indicated that the JA pathway is important for the production or transfer of the systemic signals (Li *et al.*, 2002). In an Arabidopsis–tobacco plant cluster system, it was shown that knocking out JA biosynthesis in Arabidopsis greatly compromised dodder-transmitted systemic signaling from Arabidopsis to tobacco (Hettenhausen *et al.*, 2017). Grafting in monocots is impossible, and whether JA plays a similar role in regulating systemic signals in monocots was unclear. By connecting a monocot and a dicot plant with a dodder and using Arabidopsis and maize mutants impaired in JA biosynthesis (Fig. 5), we demonstrate that JA is also important for herbivory-induced systemic signaling in monocots. Compared with angiosperms, nothing is known about SWRs in lower vascular plants. Wounding and applying the OS from the generalist insect *S. littoralis* or specialist *Strongylogaster multifasciata* induced similar levels of JA in the fern *Pteridium aquilinum* (Radhika *et al.*, 2012). In contrast, the fern *Pteris vittata* produced greater levels of oxidative burst and volatile terpenoids in response to *S. littoralis* feeding than to mechanical wounding (Imbiscuso *et al.*, 2009). In the sword fern *N. cordifolia*, we also found that W+OS treatment induced much greater levels of JA-Ile contents than did mechanical wounding treatment (Supplementary Fig. S5). Therefore, like many monocots and dicots, some fern species, though probably not all, are able to perceive certain components in the *S. litura* OS and highly activate the JA pathway. Based on the fact that the JA pathway plays an important role in controlling systemic signaling in angiosperms and the systemic signals from sword fern could be transferred and activate the defensive responses in dodder-bridged tobacco, we propose the scenario that under insect feeding stress, ferns are able to perceive insect OS and strongly elevate their JA-Ile contents, resulting in activation of SWRs, protecting ferns from subsequent insect attack.

Recent studies have revealed that the JA pathway emerged in the common ancestor of extant land plants (Monte *et al.*, 2018). The genome sequencing effort on the moss *Physcomitrella patens* and liverwort *Marchantia polymorpha* indicated the existence of *COI1*, *JAZ*, *MYC*, *NINJA*, and *TPL*, which are the core JA signaling components (Han, 2017), although *P. patens* and *M. polymorpha* can synthesize OPDA (12-oxo-phytodienoic acid, a JA precursor) but cannot synthesize JA or JA-Ile, as their genomes do not have *OPR3* or *JAR1*; instead, *M. polymorpha* uses dinor-OPDA (dinor-*cis*-OPDA and dinor-*iso*-OPDA), which bind MpCOI1, to activate downstream signaling (Monte *et al.*, 2018). Studies based on molecular data and fossil calibration have shown that arthropods colonized land almost synchronously with land plants (Rota-Stabelli *et al.*, 2013; Morris *et al.*, 2018). It is possible that the arms race between the ancestors of land plants and arthropods led to the evolution of the JA pathway, which is required for controlling the biosynthesis of defensive secondary metabolites against arthropods. SWR probably also evolved in the early land plants for defense against the motile arthropod herbivores. It would be interesting to investigate whether in the oldest embryophytes, namely bryophytes (liverworts, mosses, and hornworts), systemic signaling still exists, and, if so, does wounding-/herbivory-induced systemic signaling also require certain JAs. This study also highlights the function of dodder in mediating ecologically meaningful interplant communications among very broad ranges of host species during insect attack. Whether other types of systemic signaling induced by abiotic and biotic stresses, such as drought and pathogen infection, are also evolutionarily conserved among vascular plants is also worthwhile studying.

## Supplementary data

The following supplementary data are available at *JXB* online.

Fig. S1. Dodder *C. campe*stris is able to simultaneously connect certain dicots and monocots.

Fig. S2. Dodder *C. campestris* conveys simulated herbivory-induced systemic signals from maize to Arabidopsis and from maize to spring onion.

Fig. S3. Heatmap depicting the changes of relative transcript levels of genes involved in defense in maize after simulated herbivory treatment on Arabidopsis and spring onion.

Fig. S4. Dodder *C. campestris* is able to simultaneously connect tobacco and sword fern.

Fig. S5. Levels of JA and JA-Ile in wounding- and simulated herbivory-treated sword fern leaves.

Table S1. DEGs in the signal recipient maize plants in response to simulated herbivory-induced systemic signals from Arabidopsis to maize.

Table S2. DEGs in the signal recipient maize plants in response to simulated herbivory-induced systemic signals from spring onion to maize.

Table S3. The commonly regulated DEGs in response to simulated herbivory-induced systemic signals in the maize of plant clusters Arabidopsis–maize and spring onion–maize.

Table S4. The GO enrichment of biological process of the co-regulated DEGs.

Table S5. The relative expression levels of co-regulated DEGs in stress-related phytohormone pathways.

Table S6. The expression levels of genes involved in benzoxazinoid biosynthesis in the maize of plant clusters Arabidopsis–maize and spring onion–maize.

erab349_suppl_Supplementary_Figures

erab349_suppl_Supplementary_Tables

## Data Availability

All the transcriptome data can be accessed at the National Genomics Data Center (https://bigd.big.ac.cn/) under BioProject ID PRJCA003331.
