# Supplementary material for: Herbivory-induced systemic signals are likely to be evolutionarily conserved in euphyllophytes
Source: J Exp Bot. 2021 Jul 22;72(20):7274–84. doi: 10.1093/jxb/erab349 (PMC8547156; doi:10.1093/jxb/erab349)
Supplement: erab349_suppl_Supplementary_Figures [file erab349_suppl_supplementary_figures.pdf]

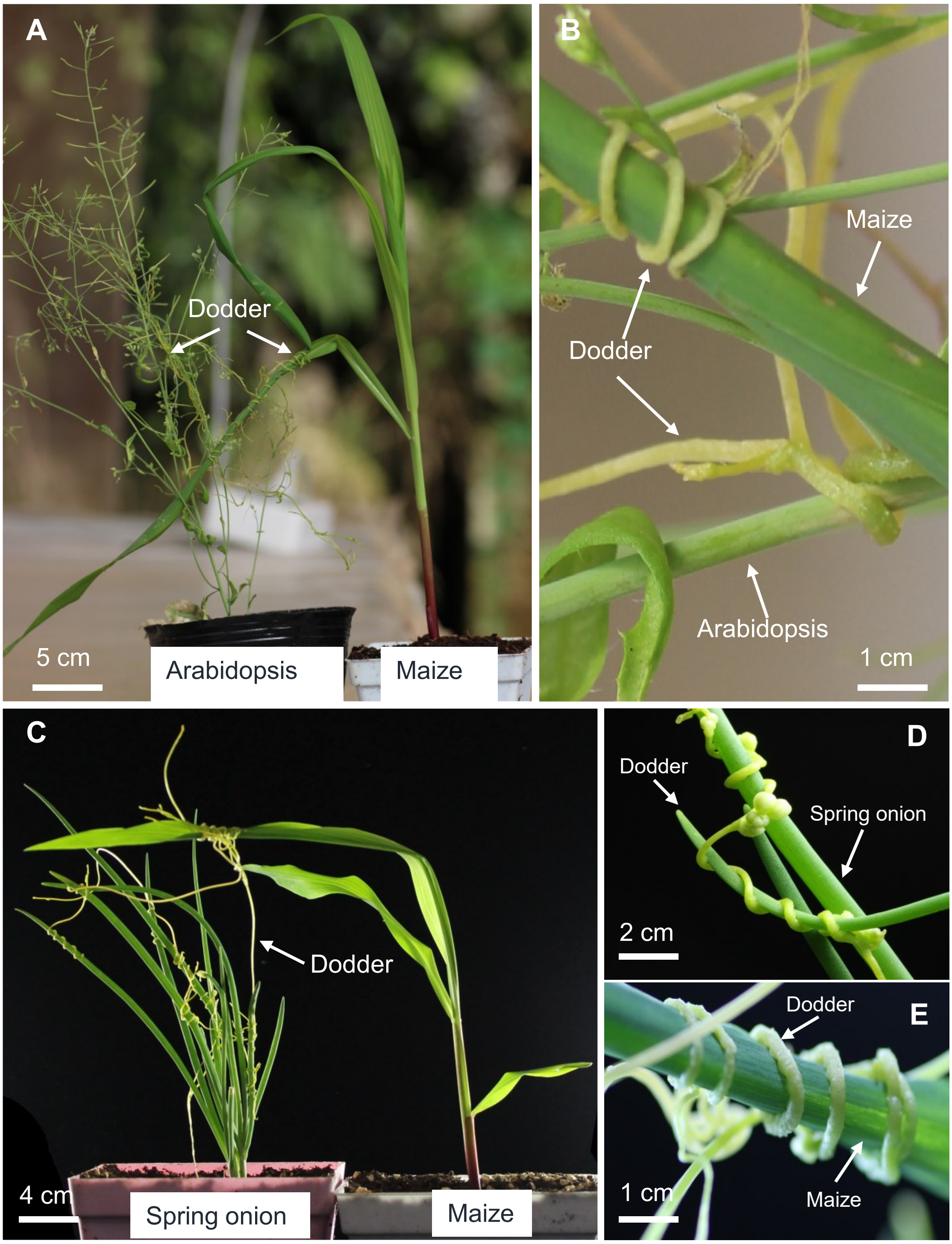

Fig. S1 Dodder *C. campestris* is able to simultaneously parasitize certain dicots and monocots. **(A)** Photograph of an Arabidopsis~maize plant cluster: Arabidopsis and maize plant were simultaneously parasitized by a *C. campestris* parasite. **(B)** Close views of the parasitized Arabidopsis and maize plant. **(C)** Photograph of a spring onion~maize plant cluster. **(D)** Close views of the parasitized spring. **(E)** Close views of the parasitized maize plant.

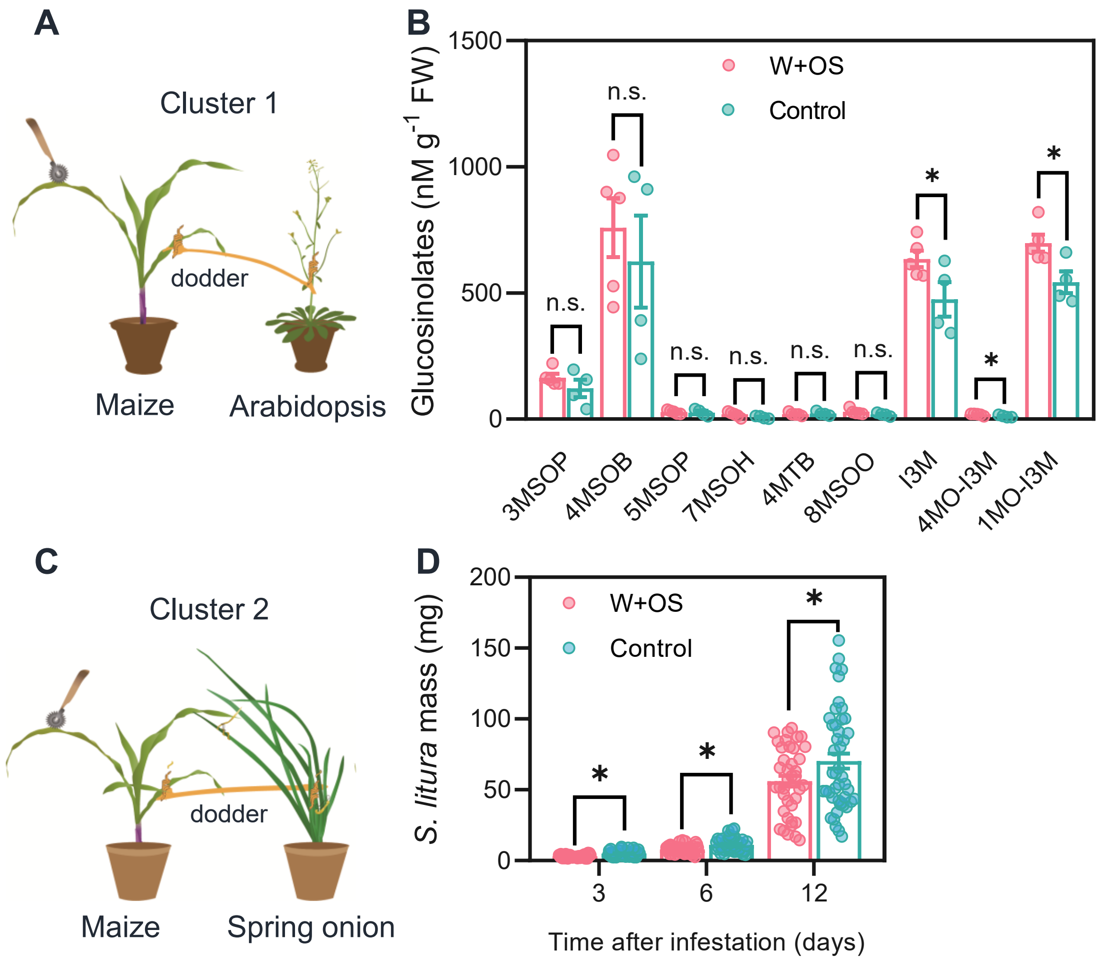

Fig. S2 Dodder *C. campestris* conveys simulated herbivory-induced systemic signals from maize to Arabidopsis and from maize to spring onion. **(A)** The schematics of Cluster 1 (maize~Arabidopsis). **(B)** The levels of glucosinolates in Arabidopsis of Cluster 1. In Cluster 1, the signal sender plants (maize) were treated with W+OS or untreated (control), after 72 h, the receiver plants Arabidopsis were harvested for quantification of glucosinolates (n = 5). **(C)** The schematics of Cluster 2 (maize~spring onion). **(D)** The growth of *S. litura* on spring onion plants in Cluster 2. In Cluster 2, maize plants were treated with W+OS. After 3 days, the spring onion plants were infested with *S. litura* larvae, and their masses were recorded over time (n = 39 to 60). Data are means ± SE, and the asterisks indicate significant differences (**P* < 0.05, *t*-test; n.s. = no significance).

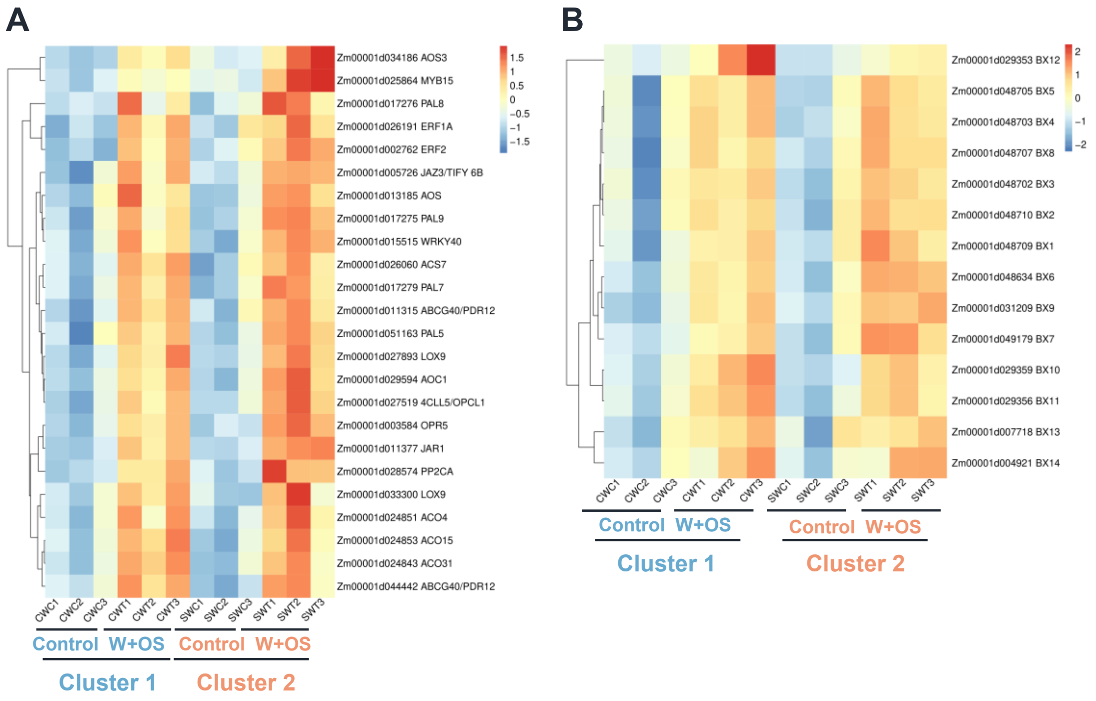

Fig. S3 Simulated herbivory elicits systemic changes gene levels in dodder-connected different hosts. In Cluster 1 (maize~Arabidopsis) and Cluster 2 (maize~spring onion), the "signal sender" plants (Arabidopsis and spring onion) were treated with W+OS treatment or not treated (control), and after 3 h the "signal receiver" plants (three replicates) were harvested for the RNA-seq. Heatmap analysis of the relative expression levels of DEGs involved in jasmonic acid, ethylene, salicylic acid, and abscisic acid pathways **(A)** and of genes involved in benzoxazinoids biosynthesis **(B)** in maize plants.

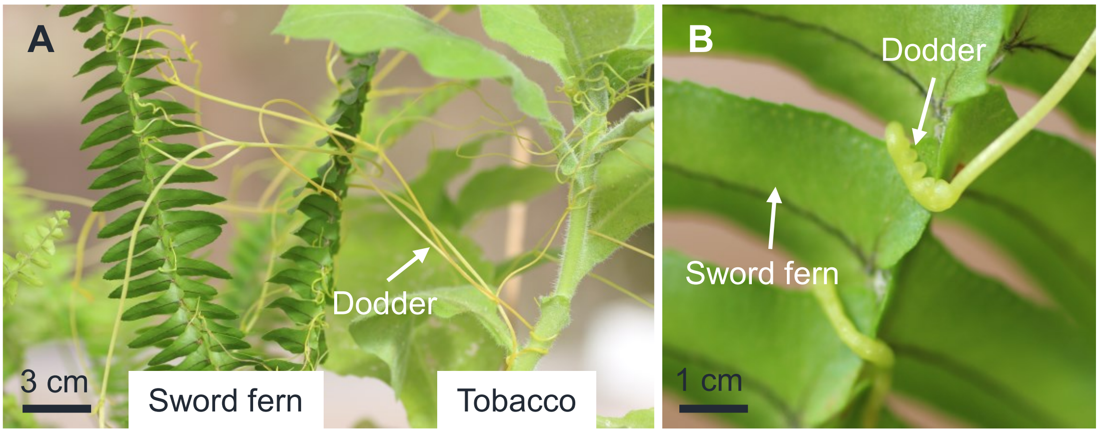

Fig. S4 Dodder *C. campestris* is able to simultaneously parasitize certain dicots and sword fern. **(A)** Photograph of a sword fern~tobacco plant cluster. Sword fern and tobacco were simultaneously parasitized by *C. campestris* parasite. **(B)** Close views of the parasitized sword fern.

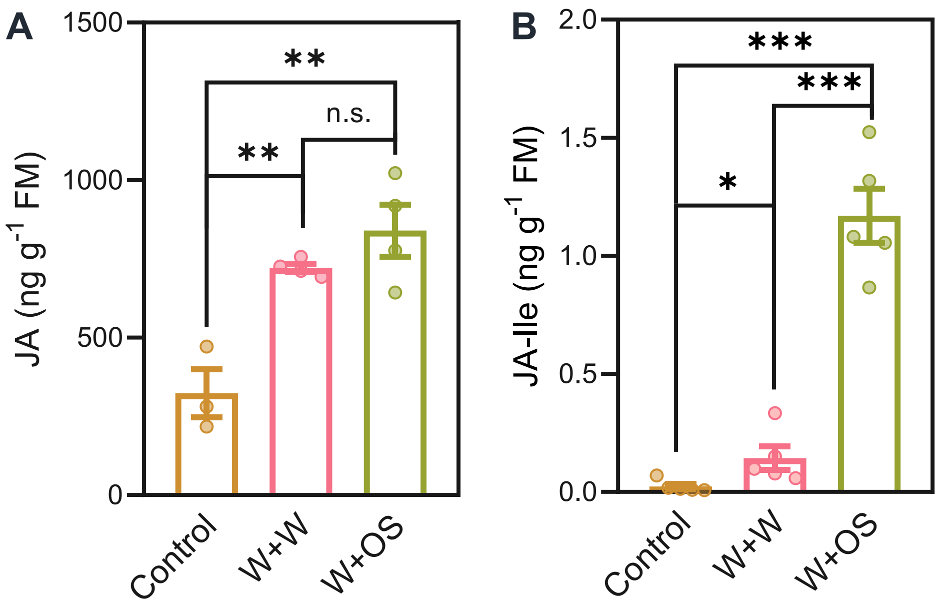

Fig. S5 Levels of JA and JA-lle in wounding- and W+OS-treated in sword fern leaves. Sword fern leaves were treated with wounded with a pattern wheel and water or *S. litura* OS were applied to wounds (W+W and W+OS, respectively); untreated plants served as controls. The leaves were harvested 1 h after treatment for determination of the JA **(A)** and JA-lle **(B)** levels (n = 5). Data are means ± SE, and the asterisks indicate significant differences (**P* < 0.05, ***P* < 0.01, and ****P* < 0.001, *t*-test; n.s. = no significance).
